# WGCNA Identifies a Comprehensive and Dynamic Gene Co-Expression Network That Associates with Smut Resistance in Sugarcane

**DOI:** 10.3390/ijms231810770

**Published:** 2022-09-15

**Authors:** Qibin Wu, Yong-Bao Pan, Yachun Su, Wenhui Zou, Fu Xu, Tingting Sun, Michael P. Grisham, Shaolin Yang, Liping Xu, Youxiong Que

**Affiliations:** 1 Key Laboratory of Sugarcane Biology and Genetic Breeding, Ministry of Agriculture and Rural Affairs, National Engineering Research Center for Sugarcane, Fujian Agriculture and Forestry University, Fuzhou 350002, China; 2 USDA-ARS, Southeast Area, Sugarcane Research Unit, Houma, LA 70360, USA; 3 Yunnan Key Laboratory of Sugarcane Genetic Improvement, Sugarcane Research Institute, Yunnan Academy of Agricultural Sciences, Kaiyuan 661600, China

**Keywords:** sugarcane, smut resistance, gene co-expression network, hub genes, WGCNA

## Abstract

Sugarcane smut is a major fungal disease caused by *Sporisorium scitamineum*, which seriously reduces the yield and quality of sugarcane. In this study, 36 transcriptome data were collected from two sugarcane genotypes, YT93-159 (resistant) and ROC22 (susceptible) upon *S. scitamineum* infection. Data analysis revealed 20,273 (12,659 up-regulated and 7614 down-regulated) and 11,897 (7806 up-regulated and 4091 down-regulated) differentially expressed genes (DEGs) in YT93-159 and ROC22, respectively. A co-expression network was then constructed by weighted gene co-expression network analysis (WGCNA), which identified 5010 DEGs in 15 co-expressed gene modules. Four of the 15 modules, namely, Skyblue, Salmon, Darkorange, and Grey60, were significantly associated with smut resistance. The GO and KEGG enrichment analyses indicated that the DEGs involving in these four modules could be enriched in stress-related metabolic pathways, such as MAPK and hormone signal transduction, plant-pathogen interaction, amino acid metabolism, glutathione metabolism, and flavonoid, and phenylpropanoid biosynthesis. In total, 38 hub genes, including six from the Skyblue module, four from the Salmon module, 12 from the Darkorange module, and 16 from the Grey60 module, were screened as candidate hub genes by calculating gene connectivity in the corresponding network. Only 30 hub genes were amplifiable with RT-qPCR, of which 27 were up-regulated upon *S. scitamineum* infection. The results were consistent with the trend of gene expression in RNA-Seq, suggesting their positive roles in smut resistance. Interestingly, the expression levels of *AOX*, *Cyb5*, and *LAC* were higher in ROC22 than in YT93-159, indicating these three genes may act as negative regulators in response to *S. scitamineum* infection. This study revealed the transcriptome dynamics in sugarcane challenged by *S. scitamineum* infection and provided gene targets for smut resistance breeding in sugarcane.

## 1. Introduction

Sugarcane (*Saccharum* spp. hybrids) is planted in more than 120 countries [1] and accounts for approximately 80% of the total sugar in the world and more than 90% in China [2]. Sugarcane smut caused by *Sporisorium scitamineum* is a worldwide fungal disease [3]. Cultivating smut-resistant varieties is the most economical and effective measure to prevent and control this disease [3,4,5]. However, sugarcane is an allopolyploid plant with a complex genetic background and an extremely low (about 1/300,000) recombination rate of excellent genes. It takes about 12 years to breed a new sugarcane variety for commercial production by traditional cross-breeding methods [6]. In addition, the smut resistance trait is determined by multiple genes [7]. It is thus of great significance to mine and identify molecular markers and key genes associated with smut resistance in sugarcane.

Compared with traditional molecular biology techniques, high-throughput bioinformatics methods can be used to locate genes of interest more quickly and easily [8,9]. The genome of a wild sugarcane species *Saccharum spontaneum* (*sspon_v201901030*) [10] has been sequenced, which provides a reference dataset for mining useful genetic information from massive biological data. Besides, the emergence of new network analysis methods has also resolved some problems of the traditional analysis methods to compare two samples with massive biological data. Weighted gene co-expression network analysis (WGCNA) is an approach that designates modules based on the expression data of gene chips or RNA sequencing (RNA-Seq). It utilizes systems biology to understand the co-expression networks and explore the associations between genes and target traits [9]. 

At present, WGCNA has been widely used in investigating the causes of a disease [11]. Shang and Gao [12] used WGCNA to analyze the gene expression of *Arabidopsis thaliana* plants infected with powdery mildew (*Erysiphe cichoracearum*; UCSC1). They constructed a co-expressed gene network and identified a total of 35 gene modules. Fu et al. [13] constructed a gene co-expression network based on the root tip transcriptome data from *Gossypium barbadense* seedlings infected with *Verticillium dahliae* at different time points. A total of 18 modules were identified, of which five were specifically related to resistance to Verticillium wilt. Four resistance genes (*GbWRKY1*, *GbRVd*, *GhCYP-3*, and *GhWRKY70*) were screened and validated. Li et al. [14] used the WGCNA method to analyze the gene expression of rice in response to the *Magnaporthe oryzae* infection. Several hub genes, including *WRKY* transcription factor, *α-dioxygenase 1*, peroxisomal membrane protein *PEX14*, and retrotransposon protein, were screened from the co-expression network. Besides, WGCNA is also a common and useful strategy for exploring the molecular mechanism of plants responding to abiotic stresses. Examples included the responses to cold, drought, and salt in rice (*Oryza sativa*) [15], drought in potato (*Solanum tuberosum*) roots [16], flood in maize (*Zea mays*) [17], and drought in rape (*Brassica rapa*) [18]. In sugarcane, WGCNA was used to analyze the effects of arbuscular mycorrhizal (AM) fungi on growth and nutrient-related gene co-expression network [19] and the drought on responsive regulatory networks [20,21]. To date, WGCNA study has not yet been reported on sugarcane response to *S. scitamineum* infection.

The present study aimed to explore gene co-expression networks associated with smut resistance in sugarcane. Firstly, the expression of differentially expressed genes (DEGs) between two sugarcane genotypes, YT93-159 (smut-resistant) and ROC22 (smut-susceptible) was analyzed at the transcriptional level. Secondly, a co-expression network of DEGs was constructed by WGCNA, and target gene modules associated with smut resistance were screened. Thirdly, the Kyoto Encyclopedia of Genes and Genomes (KEGG) and Gene ontology (GO) analyses were conducted to reveal the main metabolic pathways involved in the target gene modules and the potential functions of these genes. The hub genes in the modules were then identified according to the connectivity of genes in the corresponding network. Lastly, the expression patterns of these selected hub genes upon *S. scitamineum* infection were verified using real-time quantitative PCR (RT-qPCR). This study is expected to construct a comprehensive and dynamic gene co-expression network that associates with smut resistance in sugarcane, which should help to set up a theoretical basis for exploring the molecular mechanism of sugarcane resistance to *S. scitamineum* and provide new genetic resources for molecular breeding of smut resistance in sugarcane. 

## 2. Results

### 2.1. Genome-Wide Transcriptome Analysis

High-throughput RNA-Seq generated a total of 402.19 GB clean reads from 36 bud samples of YT93-159 and ROC22 at six time points post *S. scitamineum*-infection (0 d, 1 d, 2 d, 3 d, 4 d, and 5 d) with three biological replicates. The data from each sample were greater than 9.71 GB with GC content ranging from 53.22% to 57.79% and Q30 base percentage more than 91.18% (Table S1). Compared with the designated *S. spontaneum* reference genome [10], the reads from each sample had an alignment efficiency between 78.55% and 86.98% (Table S1). These results indicated that the quality of the sequencing data was high and met with the requirements of the subsequent analysis.

**Figure 1:**
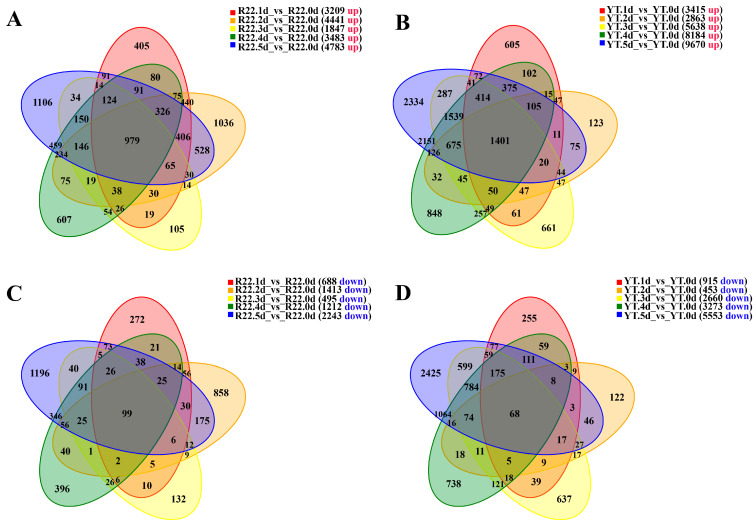
Venn plots of DEGs in ROC22 and YT93-159 post-*S. scitamineum* infection: Up-regulated DEGs in ROC22 (**A**) and YT93-159 (**B**), and down-regulated DEGs in ROC22 (**C**) and YT93-159 (**D**), respectively.

Based on the expression at different time points post *S. scitamineum* inoculation, the DEGs were identified by comparing to the control (0 d). A total of 11,897 (7806 up-regulated and 4091 down-regulated) and 20,273 (12,659 up-regulated and 7614 down-regulated) DEGs were identified in ROC22 and YT93-159, respectively (Figure 1). Among these DEGs, 979 and 1401 genes were up-regulated in ROC22 and YT93-159 at 1 d to 5 d, respectively (Figure 1A,B). In comparison with the control (0 d), 99 down-regulated genes were observed in ROC22 at all the five time points, which was not significantly different from 68 genes in YT93-159 (Figure 1C,D). The results indicated that more genes were induced to express upon *S. scitamineum* in YT93-159 than in ROC22.

### 2.2. Weighted Gene Co-Expression Network Construction and Module Identification

**Figure 2:**
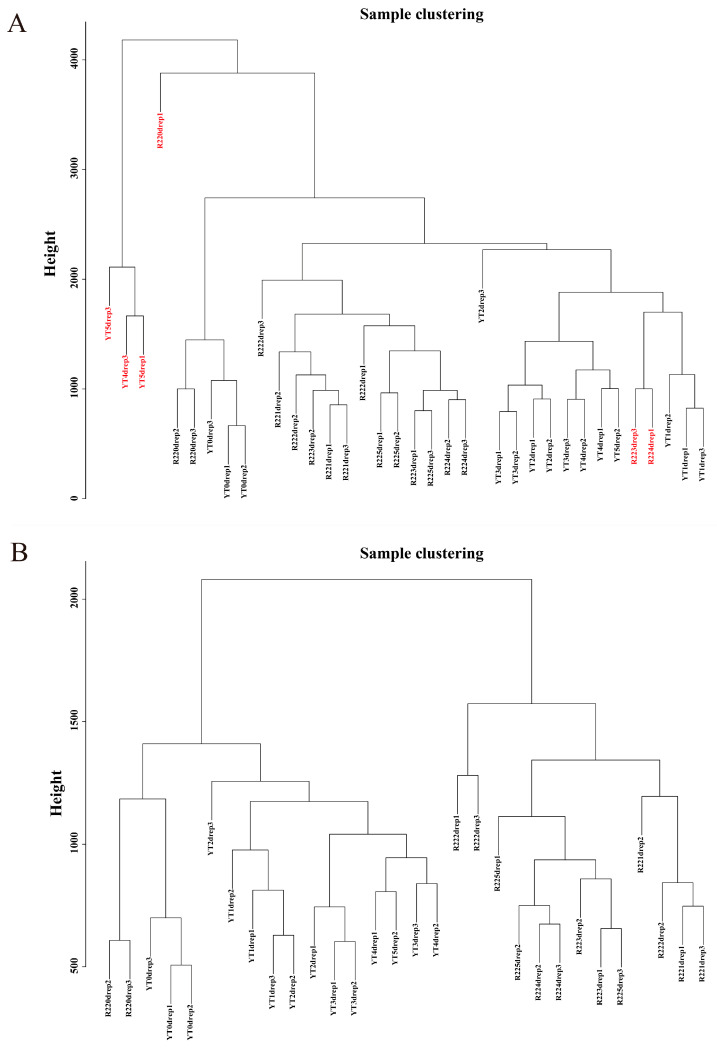
Dendrograms of samples. (**A**) A dendrogram of all 36 samples. The six outlier samples are written in red fonts. (**B**) A dendrogram of 30 samples after removing the six outliers.

**Figure 3:**
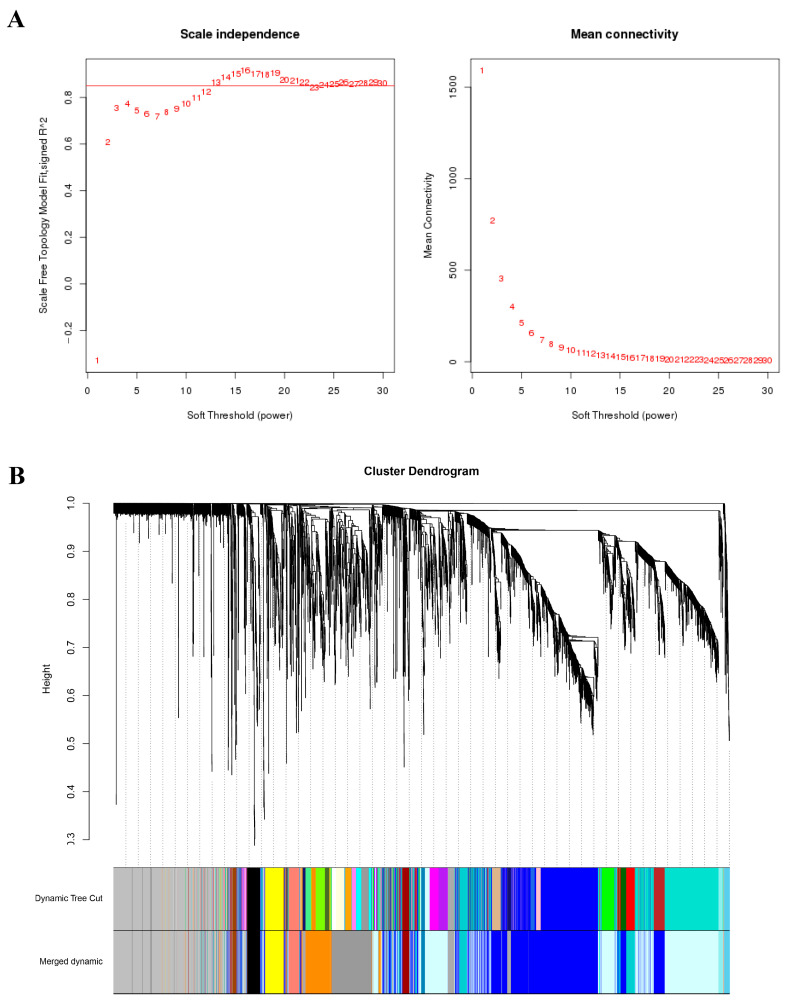
Soft threshold determination of gene co-expression network and module detection by gene cluster dendrograms. (**A**) Soft threshold determination. (**B**) Module detection by gene cluster dendrograms.

All 36 samples were clustered to calculate the correlation coefficients of expression level for each sample (Figure 2A). After removing the six outlier samples (R22.0d.rep1, R22.3d.rep3, R22.4d.rep1, YT.4d.rep3, YT.5d.rep1, and YT.5d.rep3), the clustering tree of the remaining 30 samples was shown in Figure 2B. The weight value was calculated using the function pick Soft Threshold in the WGCNA package [9], and the soft threshold β = 13 was determined when the fitting curve was close to 0.9 for the first time (Figure 3A). Then the modules with similar expression were merged by dynamic cutting tree method, and a total of 5010 DEGs in 15 co-expressed gene modules were obtained (Figure 3B). Among them, the Blue module had the largest number of DEGs (1291), of which 935 genes could be annotated. While the number of DEGs in the Paleturquoise and Steelblue modules was the least, both containing 39 genes, and among them, 28 and 34 genes can be annotated, respectively. The Grey module was a set of genes not allocated to other modules (Table S2).

**Figure 4:**
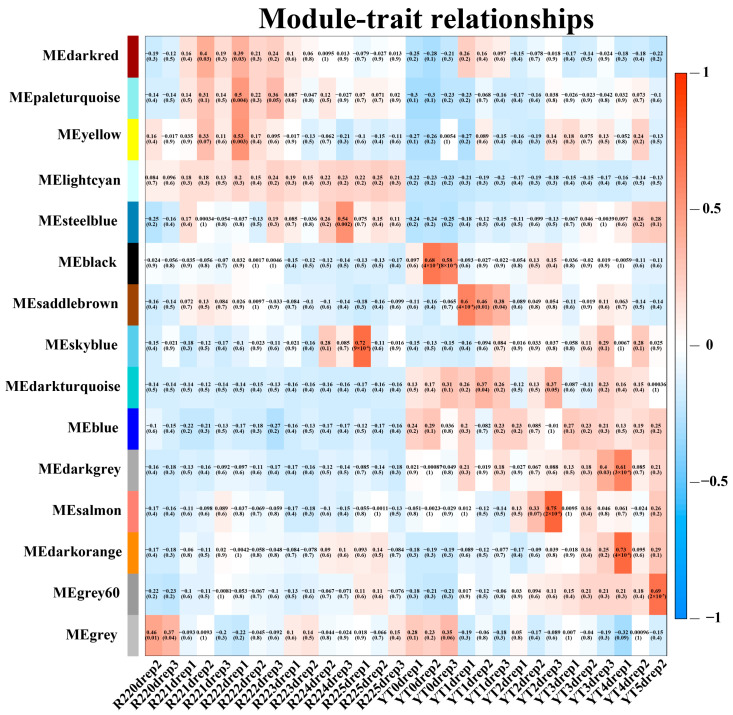
Module-trait associations revealed by Pearson correlation coefficient. The leftmost color column indicates different co-expression modules. The numbers in the figure indicate the correlation between the modules and traits, and the numbers in the parentheses are the correlation *p* value.

Correlation coefficient between modules eigengenes and traits was tested to explore the relationship between identified modules and the smut resistance. Among 15 modules, only four modules showed a significant correlation with smut resistance. The Skyblue module had a significant correlation with the smut resistance at 5 days after ROC22 inoculation with *S. scitamineum* (r^2^ = 0.72, *p* = 9 × 10^−6^). Similarly, the Grey60 modules was strongly correlated with the smut resistance at 5 days after YT93-159 inoculation with *S. scitamineum* (r^2^ = 0.69, *p* = 2 × 10^−5^). Besides, the Salmon and Darkorange modules demonstrated a significant correlation with the smut resistance at 2 days and 4 days after YT93-159 inoculation with *S. scitamineum*, respectively (r^2^ = 0.75, *p* = 2 × 10^−6^; r^2^ = 0.73, *p* = 4 × 10^−6^) (Figure 4). Therefore, the Skyblue, Salmon, Darkorange and Grey60 modules were selected as target gene modules.

### 2.3. GO and KEGG Enrichment Analysis of Four Target Gene Modules

**Figure 5:**
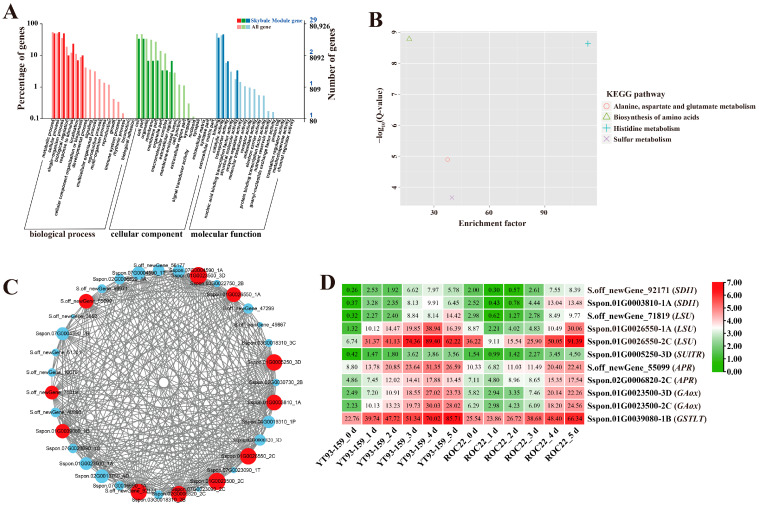
GO enrichment, KEGG enrichment, gene co-expression network, and expression patterns of hub genes in the Skyblue module. (**A**) GO functional enrichment analysis. (**B**) KEGG functional enrichment analysis. (**C**) A gene co-expression network and hub genes. The red represents hub gene, and genes with a higher connectivity in the corresponding network are shown in larger circles. (**D**) The expression patterns of hub genes. YT93-159_0 d to _5 d and ROC22_0 d to _5 d represent YT93-159 and ROC22 at 0 d to 5 d post-*S. scitamineum* inoculation, respectively.

**Figure 6:**
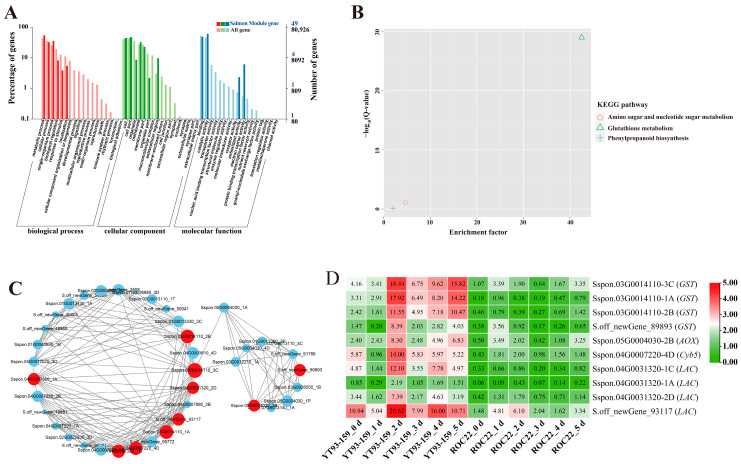
GO enrichment, KEGG enrichment, gene co-expression network, and expression patterns of hub genes in the Salmon module. (**A**) GO functional enrichment analysis. (**B**) KEGG functional enrichment analysis. (**C**) A gene co-expression network and hub genes. The red represents hub gene, and genes with a higher connectivity in the corresponding network are shown in larger circles. (**D**) Expression patterns of hub genes. YT93-159_0 d to _5 d and ROC22_0 d to _5 d represent YT93-159 and ROC22 at 0 d to 5 d post-*S. scitamineum* inoculation, respectively.

**Figure 7:**
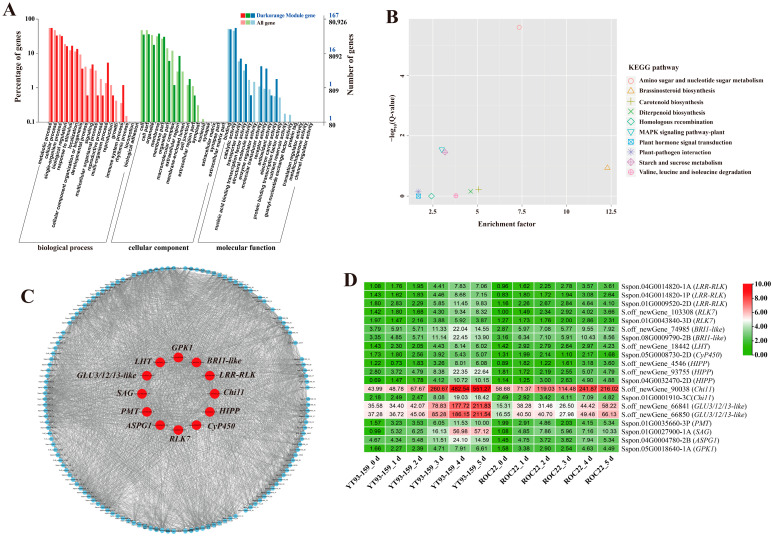
GO enrichment, KEGG enrichment, gene co-expression network and expression patterns of hub genes in the Darkorange module. (**A**) GO functional enrichment analysis. (**B**) KEGG functional enrichment analysis. (**C**) A gene co-expression network and hub genes. The red represents hub gene. Genes with a higher connectivity in the corresponding network are shown in larger circles. (**D**) Expression patterns of hub genes. YT93-159_0 d to _5 d and ROC22_0 d to _5 d represent YT93-159 and ROC22 at 0 d to 5 d post-*S. scitamineum* inoculation, respectively.

**Figure 8:**
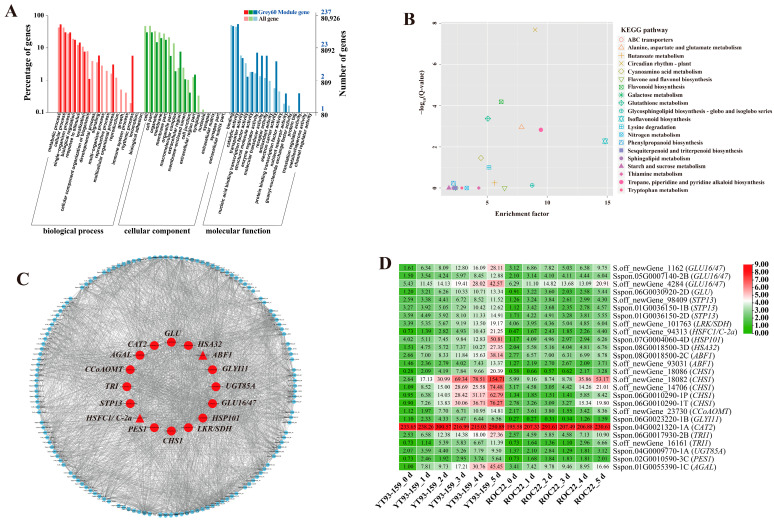
GO enrichment, KEGG enrichment, gene co-expression network and expression patterns of hub genes in the Grey60 module. (**A**) GO functional enrichment analysis. (**B**) KEGG functional enrichment analysis. (**C**) A gene co-expression network and hub genes. The red represents hub gene, the triangle represents transcription factor, and genes with a higher connectivity in the corresponding network are shown in larger circles. (**D**) Expression patterns of hub genes by transcription measured. YT93-159_0 d to _5 d and ROC22_0 d to _5 d represent YT93-159 and ROC22 at 0 d to 5 d post-*S. scitamineum* inoculation, respectively.

For the four target gene modules, the three most significantly enriched GO terms in the biological process (BP), cellular component (CC), and molecular function (MF) groups were metabolic process, cellular process, and single-organism process in the BP category, cell, cell part and organelles in the CC category, and binding, catalytic activity, and transporter activity in the MF category (Figure 5A, Figure 6A, Figure 7A and Figure 8A). The Skyblue module could be enriched in cellular response to external stimulus (GO: 0071496), glutathione transferase activity (GO: 0004364), and asparagine synthase activity (GO: 0004066). The Salmon module was enriched into glutathione transferase activity (GO: 0004364), chitinase activity (GO: 0004568), oxidoreductase activity (GO: 0016491) and lignin catabolic process (GO: 0046274). The Darkorange module was enriched into chitinase activity (GO: 0004568), transmembrane transporter activity (GO: 0022875), defense response (GO: 0006952), and transmembrane receptor protein serine/threonine kinase activity (GO: 0004675). The Grey60 module was enriched in glutathione transferase activity (GO: 0004364), lignin catabolic process (GO: 0046274), defense response (GO: 0006952), cellular response to external stimulus (GO: 0071496), abscisic acid-activated signaling pathway (GO: 0009738), and aromatic compound biosynthetic process (GO: 0019438) (Table S3). Overall, the DEGs in the four target gene modules could be enriched in the metabolic pathways related to stress resistance, such as cellular response to stress, response to external stimulus, oxidoreductase activity, glutathione transferase activity, and chitinase activity.

The KEGG enrichment analysis of DEGs in the four target gene modules identified 4, 3, 10, and 34 enriched KEGG pathways, respectively (Figure 5B, Figure 6B, Figure 7B and Figure 8B). The Skyblue module was mainly enriched in three amino acid metabolic pathways, including amino acid biosynthesis (ko01230), histidine metabolism (ko00340), and alanine, aspartic acid, and glutamate metabolism (ko00250). The Salmon module was enriched in glutathione metabolism (ko00480), amino sugar and nucleotide sugar metabolism (ko00520), and phenylpropanoid biosynthesis (ko00940). The Darkorange and Grey60 modules were mainly enriched in amino sugar and nucleotide sugar metabolism (ko00520), MAPK signaling pathway-plant (ko04016), starch and sucrose metabolism (ko00500), plant-pathogen interaction (ko04626), and plant hormone signal transduction (ko04075). In addition, the Grey60 module was also enriched in circadian rhythm-plant (ko04712), flavonoid biosynthesis (ko00941), glutathione metabolism (ko00480), phenylpropanoid biosynthesis (ko00940), and other metabolic pathways (Table S4). In general, the DEGs in the four target gene modules were enriched in stress-related metabolic pathways such as MAPK and hormone signal transduction, starch and sucrose metabolism, plant-pathogen interaction, amino acid metabolic, glutathione metabolism, flavonoid and phenylpropanoid biosynthesis.

### 2.4. Screening and Functional Annotation of Candidate Hub Genes

A total of 38 genes with high connectivity in four target gene modules were selected as candidate hub genes, including six genes in the Skyblue module, four genes in the Salmon module, 12 genes in the Darkorange module and 16 genes in the Grey60 module (Figure 5C, Figure 6C, Figure 7C and Figure 8C). The NCBI and TAIR databases were used to annotate the homologous functions of the 38 hub genes in *Arabidopsis*, most of which were related to plant stress resistance. They included chitinase 11 (*Chi11*) [22,23,24,25,26], beta-1, 3-glucosidase (*GLU*) [27,28,29,30,31,32,33], glutathione S-transferase (*GST*) [34,35,36,37,38], chalcone synthase 1 (*CHS1*) [39], caffeoyl coenzyme A O-methyltransferase (*CCoAOMT*) [40], heavy metal-associated isoprenylated plant protein (*HIPP*) [41,42], leucine-rich repeat receptor-like protein kinase (*LRR-RLK*) [43,44,45], laccase (*LAC*) [46,47,48,49,50,51,52], ABA responsive element binding factor 1 (*ABF1*) [53], and so on (Table S6).

### 2.5. Validation of Hub Genes by RT-qPCR

**Figure 9:**
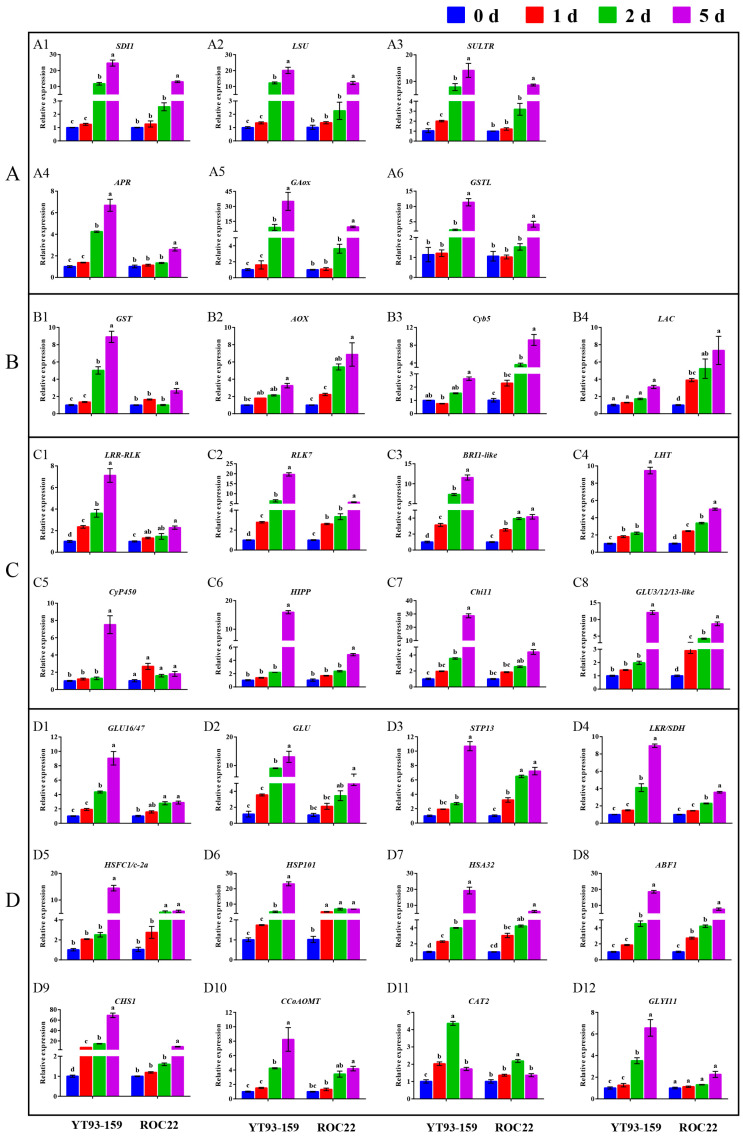
Relative expression of 30 hub genes in sugarcane after inoculation with *S. scitamineum* by RT-qPCR. (**A**) Skyblue module. (**B**) Salmon module. (**C**) Darkorange module. (**D**) Grey60 module. 0 d (blue), 1 d (red), 2 d (green), and 5 d (purple) for YT93-159 and ROC22 at 0 d, 1 d, 2 d, and 5 d post-*S. scitamineum* inoculation, respectively. All data points are mean ± standard error (*n* = 3). Bars superscripted by different lowercase letters indicate significant differences at 5% level (*p* ≤ 0.05). A1, *SDI1* (sulfur deficiency-induced 1); A2, *LSU* (LOW SULPHUR UPREGULATED); A3, *SULTR* (sulfate transporter); A4, *APR* (adenosine 5’-phosphate sulfate reductase); A5, *GAox* (gibberellin oxidase); A6, *GSTL* (glutathione S-transferase lambda); B1, *GST* (glutathione S-transferase); B2, *AOX* (alternative oxidase); B3, *Gyb5* (cytochrome b5); B4, *LAC* (laccase); C1, *LRR-RLK* (leucine-rich repeat receptor-like protein kinase); C2, *RLK7* (receptor-like kinase 7); C3, *BRI1-like* (brassinosteroid insensitive 1-like); C4, *LHT* (lysine histidine transporter); C5, *CyP450* (cytochrome P450); C6, *HIPP* (heavy metal-associated isoprenylated plant protein); C7, *Chi11* (chitinase 11); C8, *GLU 3/12/13-like* (beta-glucosidase 3/12/13-like); D1, *GLU16/47* (beta-glucosidase 16/47); D2, *GLU* (beta-glucosidase); D3, *STP13* (sugar transport protein 13); D4, *LKR/SDH* (lysine-ketoglutarate reductase/saccharopine dehydrogenase); D5, *HSFC1/C-2a* (heat shock transcription factor C1/C-2a); D6, *HSP101* (heat shock protein 101); D7, *HSA32* (heat-stress-associated 32); D8, *ABF1* (ABA responsive element binding factor 1); D9, *CHS1* (chalcone synthase 1); D10, *CCoAOMT* (caffeoyl coenzyme A O-methyltransferase); D11, *CAT2* (catalase 2); D12, *GLYI11* (glyoxalase I 11).

Only 30 hub genes had successful RT-qPCR amplification. Twenty-seven hub genes can be induced and up-regulated by *S. scitamineum* infection. The gene expression levels of these genes were significantly higher in YT93-159 than ROC22 (Figure 9 and Table S8), which was consistent with the results from RNA-Seq (Figure 5D, Figure 6D, Figure 7D and Figure 8D and Table S7) suggesting the positive roles of these genes in smut resistance. However, the expression levels of *AOX*, *Cyb5* and *LAC* genes were inconsistent with the trend of gene expression in RNA-Seq (Figure 6D) and were higher in ROC22 than YT93-159, suggesting that these genes may act as negative regulators in response to *S. scitamineum* infection (Figure 9B).

## 3. Discussion

Cultivating smut-resistant varieties is the most economical and effective measure to prevent and control this disease in sugarcane industry [3,4,5]. Mining key candidate genes associated with smut resistance can provide target gene resources for molecular breeding for smut resistance in sugarcane. Previous studies in transcriptome [54,55,56,57,58,59,60] and proteome [61,62,63,64,65] on sugarcane infected by *S. scitamineum* offered new insights as to how *S. scitamineum* infection might influence the differential expression of genes and proteins. These studies also revealed that the mechanism of smut resistance was extremely complex and involved in almost all aspects of biological activities [5]. In this study, WGCNA was applied to construct a co-expression network of weighted genes associated with smut-resistant traits based on multivariate transcriptome dataset from plants of two sugarcane genotypes, YT93-159 (resistant) and ROC22 (susceptible) that were infected by *S. scitamineum*. A total of 15 gene modules were identified, of which four were significantly associated with smut resistance. Interestingly, 38 candidate hub genes with high connectivity were successfully mined (Figure 5C, Figure 6C, Figure 7C and Figure 8C), of which 30 were induced and expressed upon *S. scitamineum* infection (Figure 9). These hub genes may play an important role in sugarcane’s response to *S. scitamineum* infection through sulfur metabolism, reactive oxygen species (ROS) scavenging mechanism, plant immune response, phenylpropanoid metabolism, brassinosteroid signaling, and other resistance-associated metabolic pathways. Sulfur, as an essential macronutrient, plays a crucial role in plant growth and development [66]. Sulfur deficiency can lead to amino acid imbalance and impaired protein synthesis [67]. Aarabi et al. [68] reported that under sulfur deficiency condition, sulfur deficiency induced 1 (SDI1) and SDI2 acted as major repressors controlling glucosinolate (GSL) biosynthesis in *Arabidopsis* at both transcript and metabolite levels. Garcia-Molina et al. [69] showed that the expression of LOW SULPHUR UPREGULATED (*LSU*) in *Arabidopsis* was up-regulated under nutrient depletion or salt stress. Adenosine 5’-phosphate sulfate reductase (APR) was the key rate-limiting enzyme in the sulfur assimilation process and was mainly regulated by transcription factors in response to sulfur availability and environmental stimuli [70]. Sulfate transporter (SULTR) was a carrier protein required for active sulfate transport, and an essential protein for absorption and transportation of sulfur in plants [71]. Ding et al. [71] identified 28 *GmSULTR* genes from soybean genome and confirmed that *GmSULTR1;2b* played an important role in sulfur absorption to improve plant tolerance to sulfur deficiency. In this study, *SDI1*, *LSU*, *SULTR* and *APR* genes in the Skyblue module responded to *S. scitamineum* infection by gradually increased levels of gene expression that reached the peak at 5 d. The expression levels were higher in YT93-159 than ROC22 (Figure 9A), indicating that these four genes may involve in the sulfur metabolism pathway in response to *S. scitamineum* infection.

Catalase (E.C.1.11.1.6; CAT), a heme-containing tetramer enzyme mostly localized in peroxisomes, is an efficient scavenger of ROS [72]. The main function of CAT is to scavenge H_2_O_2_, maintain the stability of ROS levels in plants to avoid oxidative damage caused by various biotic and abiotic stresses [73,74]. Su et al. [75] found a sugarcane catalase gene *ScCAT1* that was up-regulated by varying degrees under *S. scitamineum* infection and abiotic stresses such as SA, ABA, MeJA, H_2_O_2_, drought, salt, and heavy metals. Similarly, Sun et al. [76] reported that *ScCAT2* played a positive role in immune responses during sugarcane-*S. scitamineum* interactions, as well as in salt, drought, and cold stresses. Glutathione S-transferases (GSTs) are a group of isozymes widely existing in plants with multiple physiological functions [77]. GST catalyzes the conjugation of tripeptide (γ-Glu-Cys-Gly) glutathione (GSH) to various electrophiles [78] and plays a crucial role in the detoxification of xenobiotics and stress metabolism [35]. In cotton, the expression of *GaGSTF9* gene can enhance the resistance of *Gossypium arboreum* to Verticillium wilt [36]. Overexpression of *GhGST3* gene could effectively improve the resistance of *Arabidopsis* plants to Verticillium wilt [37]. In this study, the *CAT2* gene in the Grey60 module was up-regulated by *S. scitamineum* infection and reached the peak at 2 d (Figure 9D12). The *GSTL* gene in the Skyblue module and the *GST* gene in the Salmon module were positively up-regulated by the stress of sugarcane smut pathogen (Figure 9A6,B1), confirming that *CAT* and *GST* may play a role in sugarcane response to *S. scitamineum* infection through the enzymatic ROS scavenging mechanism.

Pathogenesis-related (PR) proteins are specific proteins induced under pathological conditions that play an important role in plant disease resistance reaction [79]. Chitinases (E.C. 3.2.2.14; Chi) are one category of PR proteins, which can play an important role in plant defense mechanisms by catalyzing poly-chitin in the main components of the pathogen cell wall [24]. Su et al. [25,26] demonstrated that the chitinase gene was involved in sugarcane response to *S. scitamineum* infection, and its expression level was higher in smut-resistant varieties than susceptible varieties. β-1,3-glucanase (E.C. 3.2.1.39; GLU) is another typical PR protein that catalyzes the hydrolysis of β-1,3-glucans in the cell wall of most fungi [27,28]. Su et al. [32,33] revealed that three sugarcane β-1,3-glucanase genes (*ScGluA1*, *ScGluD1*, and *ScGluD2*) were involved in sugarcane response to *S. scitamineum* infection and their expression levels were higher in smut-resistant varieties than susceptible varieties. Besides, previous study showed that the co-expression of tobacco β-1,3-glucanase and chitinase gene effectively inhibited the growth of smut pathogen in sugarcane [29]. In this study, *Chi11*, *GLU3/12/13-like*, *GLU16/47*, and *GLU* genes were induced and up-regulated upon *S. scitamineum* infection (Figure 9C7,C8,D1,D2), suggesting that these genes may play a role in the interaction between sugarcane and *S. scitamineum*.

Phenylpropanoid metabolism is one of the most important metabolisms in plants with many intermediate compounds, including lignin, flavonoids, lignans, phenylpropanoid esters, and hydroxycinnamic acid amides, that are closely related to plant resistance [80]. Chalcone synthase (CHS) is a key enzyme in the synthesis of multifunctional flavonoids involving in plant resistance to biotic/abiotic stress [39]. After *S. scitamineum* inoculation, Wang et al. [81] found an up-regulated expression of *ScCHS1* gene in NCo376, a smut-resistant variety, while there was no significant change in susceptible cultivar ROC22. Caffeoyl-coenzyme A O-methyltransferase (CCoAOMT) acts as the main regulator determining the efficiency of synthesis and composition of lignin [82]. Yang et al. [40] showed that *CCoAOMT* the expression could improve wheat resistance to *Fusarium* head blight (FHB). We found in this study that the expression levels of *CHS1* and *CCoAOMT* genes increased gradually from 0 d to 5 d upon *S. scitamineum* inoculation and peaked at 5 d (Figure 9D9,9D10). The results suggest that these two genes may involve in the synthesis of flavonoids and lignin and hence enhance the resistance to sugarcane smut. Laccase (LAC), also known as a polyphenol oxidase, has a typical copper oxidase domain and belongs to an enzyme of the cuprous oxidase family [46]. The LAC is mainly involved in the metabolism of lignin, phenols and other substances, the formation of cell wall [47], and the regulation of plant growth and development [48], stress response [49], insect and disease resistance [50,51] in higher plants. Hu et al. [52] showed that cotton laccase gene *GhLac1* accelerated the reconstruction of protoplast cell wall and participated in the regulation of cotton resistance to *Verticillium dahliae*, cotton bollworm (*Helicoverpa armigera*) and cotton aphid (*Aphis gosypii*). Interestingly, in our study, the expression level of *LAC* gene in smut-susceptible variety ROC22 was higher than that in -resistant variety YT93-159 (Figure 9B4), which indicated that *LAC* gene may be involved in the metabolism of lignin and phenols as a negative regulatory gene to improve sugarcane resistance to *S. scitamineum*. 

The brassinosteroid (BR) signaling pathway not only plays an important role in plant growth and development, but also involves in plant immune defense [83]. In this study, leucine-rich repeat receptor-like protein kinase gene (*LRR-RLK*) (Figure 9C1), receptor-like kinase 7 gene (*RLK7*) (Figure 9C2) and brassinosteroid insensitive 1-like gene (*BRI1-like*) (Figure 9C3) were screened out from the Darkorange module and were up-regulated by the *S. scitamineum* infection. These genes may involve in plant BR signaling pathway and play an important role in sugarcane response to *S. scitamineum* infection, as was suggested by previous studies [43,84,85]. For example, Chen et al. [84] demonstrated that overexpression of *TaRLK1* or *TaRLK2* in a moderately susceptible wheat cultivar “Yangmai 158” could significantly enhance the resistance to powdery mildew (*Blumeria graminearum* f. sp. *tritici*). Guo et al. [43] confirmed that by silencing the cysteine-rich receptor-like kinase (CRK)-encoding gene (*TaCRK3*) significantly reduced wheat resistance to Sharp eyespot (*Rhizoctonia cerealis*) and inhibited the expression of the ethylene biosynthesis *ACO2* and other genes related to defense. Marcos et al. [85] found that 9-lipoxygenase-derived oxylipins in *A. thaliana* induced BR synthesis and signaling to activate cell wall-based responses, while a BR receptor BRI1 deletion mutant *bri1* gene conferred resistance to *Pseudomonas syringae*.

## 4. Materials and Methods

### 4.1. Plant Materials and Pathogen Stress Treatment

Two sugarcane genotypes, YT93-159 and ROC22, were planted at the Experimental Station of Fujian Agriculture and Forestry University (FAFU), Fuzhou, Fujian (longitude 119°23′ E/latitude 26°11′ N), during a natural growth season in 2018 to 2019. Cultivation and field management followed normal production protocols. Mixed spores of *S. scitamineum* were collected from a sugarcane field plot in Baise, Guangxi (longitude 106°53′–107°26′ E/latitude 23°16′–24°01′ N) and the Experimental Station of FAFU, then stored at 4 °C refrigerator after drying.

Mature sugarcane stems of YT93-159 and ROC22 with the same growth vigor were selected and inoculated with *S. scitamineum* according to Su et al. [75]. The sugarcane stems were cut into single-bud setts, soaked in water for 24 h, and then germinated for 2–3 d in a greenhouse at 32 °C, 65% relative humidity (RH). When the sugarcane buds germinated and grew to 1–2 cm long, they were syringe-inoculated with 0.5 μL suspension containing 5 × 10^6^ spores mL^−1^ in 0.01 % (*v/v*) Tween-20. The inoculated buds were cultured at 28 °C, 65% RH under conditions for 12 h light/12 h dark alternately. At least six buds from each genotype were excised with three biological replicates at the time point of 0 d (control), 1 d, 2 d, 3 d, 4 d and 5 d, then frozen in liquid nitrogen and stored at −80 °C until extraction of total RNA.

### 4.2. RNA Extraction, Illumina Sequencing and Data Analysis

Total RNA of 36 samples was extracted using Trizol reagent (Invitrogen, Carlsbad, CA, USA) following the manufacturer’s protocol. The total RNA samples were sent to Baimaike Biotechnology Co., Ltd. (Beijing, China) for cDNA library construction after qualitative and quantitative assessments on NanoDrop One (Thermo Fisher Scientific, Waltham, MA, USA). The RNA integrity (RIN) was also confirmed using an Agilent 2100 Bioanalyzer (Agilent Technologies, Palo Alto, CA, USA). The requirements for cDNA library construction and subsequent sequencing were met when the RIN was ≥7. Libraries for Illumina RNA-Seq (150 bp paired-end) were generated using the TruSeq RNA sample preparation kit (Illumina RS-122–2101, Illumina, CA, USA) and sequenced on an Illumina HiSeq2000 instrument. The raw sequencing data were deposited in National Genomics Data Center (NGDC), Beijing Institute of Genomics, Chinese Academy of Sciences, under Project PRJCA011580 with Genome Sequence Archive (GSA) number CRA008043 (https://ngdc.cncb.ac.cn/gsub/submit/gsa/subCRA012317/finishedOverview; accessed on 31 August 2022 and released on 5 September 2022).

The quality of sequencing was assessed using FastQC and Trimmomatic software [86]. Clean data were obtained by removing reads containing adapter, poly-N and low-quality reads from raw data, then mapped to the *S. spontaneum* reference genome [10] using HISAT2 [87]. The *S. spontaneum* reference genome was downloaded from EnsemblPlants (http://plants.ensembl.org/species.html; accessed on 8 March 2021). The FPKM (fragments per KB of transcript per million fragments mapped) in StringTie [88] was used to measure the level of transcript or gene expression. The DESeq R [89] package was then used to conduct differential expression analysis of samples. In this study, DEGs were identified if fold change was >1.5 and the false discovery rate (FDR) was <0.05.

### 4.3. Weighted Gene Co-Expression Network Analysis

The co-expression gene network was constructed using the WGCNA package of the R software in reference to the tutorial on WGCNA official website [9]. Firstly, clustering analysis was carried out after removing samples with low correlation, or those that cannot be clustered on the dendrogram [16]. Secondly, to meet the prerequisite of scale-free network distribution, the pick Soft Threshold function within WGCNA package was used to calculate the Soft Threshold [9], and the Threshold parameter β was selected when the fitting curve approached 0.9 for the first time. Then, according to the β value, the correlation-based association between phenotype and gene-modules was conducted to create an adjacency matrix. The adjacency matrix was further converted to a topological overlap matrix (TOM), and the gene connection network was constructed. Finally, the gene modules were identified and clustered by dynamic tree cut method based on the eigengenes (ME) of each module, and the modules with closer distances were merged into new ones. In this study, the module similarity threshold = 0.25, the expression threshold = 2, and the minimum number of genes in the module = 30 were set to divide modules.

### 4.4. Selection and Enrichment Analysis of Target Gene Modules

To further study the gene modules associated with sugarcane smut resistance, the correlation coefficients between module eigengenes and different samples were calculated. Modules with correlation coefficients close to 0.7 (r ≈ 0.7) were selected as target gene modules. Annotation of DEGs in target modules was carried out by using Blast2Go [90,91] with default parameters at each step of the pipeline. Blast2Go retrieves gene ontology (GO) terms that include biological processes, molecular functions, and cellular components. Pathway enrichment analysis of DEGs within the target modules was performed using KEGG database [92]. 

### 4.5. Screening and Functional Annotation of Hub Genes

All the DEGs from these four target modules were selected based on intramodular gene significance, edges, and nodes calculated with the WGCNA R package [9,93] (Table S5). The information was exported to Cytoscape 3.8.2 software [94] for gene-networks visualizations. The DEGs with high connectivity in the target gene modules were screened as candidate hub genes. The NCBI database was used to query related reports of these hub genes. Homologous gene functions in *Arabidopsis* were annotated with the TAIR database (https://www.arabidopsis.org; accessed on 15 September 2021).

### 4.6. Validation of Hub Genes by Real-Time Quantitative PCR Analysis

A total of 38 candidate hub genes were selected for RT-qPCR analysis to examine their expression profiles. Primer pairs of candidate hub genes were designed by Beacon Designer 8.0 (Table S9). The buds of YT93-159 and ROC22 at 0 d, 1 d, 2 d, and 5 d time points post-*S. spontaneum* inoculation were selected as materials. *GAPDH* was used as the internal reference gene [95]. The gene expression analysis of candidate hub genes was carried out with three independent biological replicates for each sample [96]. RT-qPCR was performed on ABI QuantStudio^TM^ 3 system (Thermo Fisher Scientific, Waltham, MA, USA) using the SYBR-green dye method following a thermal cycling program of “50 °C, 2 min; 95 °C, 10 min; 40 cycles of (95 °C, 15 s; 60 °C, 1 min)”. The total reaction volume was 25 µL, including 12.5 µL FastStart Universal SYBR Green PCR Master (Roche, Shanghai, China), 0.5 µL of each primer (10 µM), 1.0 µL template (10× diluted cDNA liquid), and 10.5 µL ddH_2_O. Expression levels of selected hub genes were calculated using the 2^−∆∆CT^ method [97]. Histograms were graphed by GraphPad Prism 6. Significance (*p* < 0.05) and standard error (SE) were determined by the Duncan’s new multiple range test.

## 5. Conclusions

**Figure 10:**
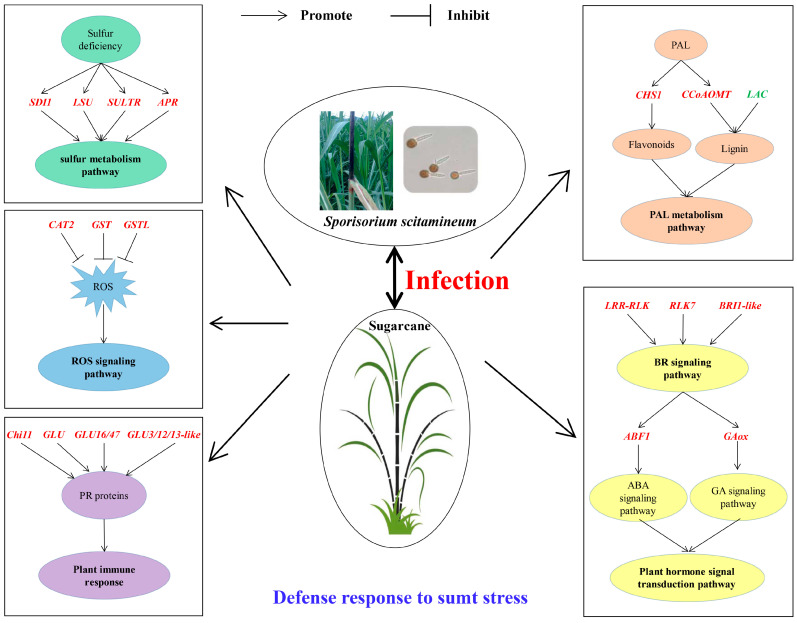
A model of molecular mechanism of sugarcane response to *S. scitamineum* infection. Positive gene regulators are written in red fonts. Negative gene regulators are written in green fonts. *SDI1*, sulfur deficiency-induced 1; *LSU*, LOW SULPHUR UPREGULATED; *SULTR*, sulfate transporter; *APR*, adenosine 5’-phosphate sulfate reductase; *CAT2*, catalase 2; *GST*, glutathione S-transferase; *GSTL*, glutathione S-transferase lambda; *Chi11*, chitinase 11; *GLU*, beta-glucosidase; *GLU16/47*, beta-glucosidase 16/47; *GLU3/12/13-like*, beta-glucosidase 3/12/13-like; *CHS1*, chalcone synthase 1; *CCoAOMT*, caffeoyl coenzyme A O-methyltransferase; *LAC*, laccase; *LRR-RLK*, leucine-rich repeat receptor-like protein kinase; *RLK7*, receptor-like kinase 7; *BRI1-like*, brassinosteroid insensitive 1-like; *ABF1*, ABA responsive element binding factor 1; *GAox*, gibberellin oxidase.

In this study, a co-expression network of weighted genes associated with sugarcane smut-resistant traits was constructed to identify 15 target modules. Four target gene modules were significantly associated with *S. scitamineum* infection. The GO and KEGG enrichment analyses revealed that the DEGs in these four target gene modules were enriched in several metabolic pathways related to stress resistance, such as MAPK and hormone signal transduction, plant-pathogen interaction, amino acid metabolism, glutathione metabolism, and flavonoid and phenylpropanoid biosynthesis. A total of 38 genes, including six from the Skyblue module, four from the Salmon module, 12 from the Darkorange module and 16 from the Grey60 module, with high connectivity in four target gene modules were screened as candidate hub genes. The RT-qPCR analysis showed that 30 genes was induced to express upon *S. scitamineum* infection. Twenty-seven genes had up-regulated expression and played a positive role in smut resistance. Interestingly, the expression levels of *AOX*, *Cyb5* and *LAC* were higher in susceptible genotype ROC22 than the resistant genotype YT93-159, indicating that these three genes may be negative regulators in sugarcane response to *S. scitamineum* infection. In addition, a potential molecular mechanism of sugarcane response to *S. scitamineum* infection was drawn (Figure 10) to illustrate what roles these hub genes may play in smut resistance in sugarcane. The results from this study benefit further understanding of the molecular mechanisms of smut resistance and provide a new gene resource for future smut resistance breeding in sugarcane.

## Data Availability

Not applicable.

## Supplementary Materials

The following supporting information can be downloaded at https://www.mdpi.com/article/10.3390/ijms231810770/s1.
